# Patterns of diabetes care in Slovenia, Croatia, Serbia, Bulgaria and Romania: An observational, non-interventional, cross-sectional study

**DOI:** 10.1007/s00508-016-1143-1

**Published:** 2016-12-08

**Authors:** Miro Cokolic, Nebojsa M Lalic, Dragan Micic, Gorana Mirosevic, Sanja Klobucar Majanovic, Ivaylo N Lefterov, Mariana Graur

**Affiliations:** 1 Department for Endocrinology and Diabetes, University Medical Center Maribor, Ljubljanska ulica 5, 2000 Maribor, Slovenia; 2 Clinic for Endocrinology, Diabetes and Metabolic Diseases, Clinical Center of Serbia, Belgrade, Serbia; 3 Department of Endocrinology, Diabetes and Metabolic Diseases “Mladen Sekso”, Clinical Hospital Centre “Sisters of Mercy”, Zagreb, Croatia; 4 Department of Internal Medicine, Division of Endocrinology, Diabetes and Metabolic Disorders, University Hospital Rijeka, Rijeka, Croatia; 5 Diagnostic Consultative Center XII, Sofia, Bulgaria; 6 Grigore T. Popa University of Medicine and Pharmacy, Iasi, Romania

**Keywords:** Type 2 diabetes, Clinical practice, HbA1c measurement rate, Glycemic control, Diabetes therapy

## Abstract

**Background:**

National guidelines for treating type 2 diabetes in the Balkans generally follow European guidelines. The current study was undertaken to estimate the rate of glycated hemoglobin (HbA1c) measurements and level of HbA1c control in diabetic patients treated in regular clinical practice settings in the Balkans and to evaluate if providing HbA1c measurements improves adherence to treatment guidelines.

**Methods:**

This cross-sectional study enrolled type 2 diabetic patients treated by 79 primary care physicians and 102 specialists. The participants were provided with HbA1c measuring devices to measure HbA1c during regular office visits and a physician survey evaluated HbA1c the results feedback. Relevant clinical, demographic, drug treatment and specialist referral data were extracted from patient charts. Descriptive statistics and stepwise multivariate regression analysis were used.

**Results:**

Among 1853 patients included (average age 63.5 ± 10.7 years, 51% male) the average diabetes duration was 8.9 ± 7.1 years, 40% of patients had HbA1c measured every 6 months and 34% every 12 months (or less frequently). The rate of 6‑month measurement was higher among specialists (43%) vs. primary care physicians (32%, *p* < 0.01). The average HbA1c was 7.3 ± 1.5 and 35% of patients achieved the target HbA1c level of < 6.5%. Metformin monotherapy was prescribed to 28% of patients and metformin + sulphonylurea to 23%, 55% of patients on metformin monotherapy and 32% of patients on dual therapy metformin + sulphonylurea achieved the target HbA1c < 6.5%. Treatment remained unchanged in 91% and was stepped up in only 7.2% of patients. Physicians were not surprised (in 79% of patients) or were pleasantly surprised (in 11%) by the HbA1c results at the time of visit. Average diabetes duration and patient use of home glucometers were associated with the level of disease control.

**Conclusions:**

The rates of HbA1c measurements remain low in the Balkans, although higher among specialists. Over 60% of patients, mostly treated with traditional oral antidiabetics did not achieve disease control. Providing convenient HbA1c measurement devices was not associated with a marked change in diabetes management. Future research is needed to evaluate the impact of these treatment patterns on long-term outcomes and costs to society.

## Introduction

Diabetes mellitus (DM) is a chronic disease that requires continuous medical care and patient self-management, education and support in order to prevent acute complications and reduce the risk of long-term complications. It is considered one of the greatest health challenges of the twenty-first century. In 2013 DM caused 5.1 million deaths [1] and a person dies from diabetes every 6 s [1]. According to the International Diabetes Federation (IDF), 8.3% of adults have diabetes, and the number of people living with diabetes is expected to rise from 382 million in 2013 to 592 million in less than 25 years [1]. Type 2 DM accounts for 90% of all diabetes cases [2]. The 2006 World Health Organization (WHO) diagnostic criteria for diabetes are fasting plasma glucose ≥7.0 mmol/l (126 mg/dl) or 2‑h plasma glucose ≥11.1 mmol/l (200 mg/dl) [3]. In 2009, an international expert committee including representatives of the American Diabetes Association (ADA), the IDF and the European Association for the Study of Diabetes (EASD) recommended the use of the HbA1c test to diagnose diabetes, with a threshold of ≥6.5% (48 mmol/mol) and the ADA adopted this criterion in 2010. Furthermore, the two most influential large-scale clinical trials of diabetes therapeutic regimens, the Diabetes Control and Complications Trial (DCCT) and the United Kingdom Prospective Diabetes Study (UKPDS), have shown that improving HbA1c levels slows the development and progression of eye, kidney, and nerve complications in both type 1 and type 2 DM [4–6]. There are two primary techniques available to assess the effectiveness of the glycemic control management plan: blood glucose and HbA1c monitoring [7]. The most abundant minor hemoglobin component is HbA1c and it is formed by the chemical condensation of hemoglobin and glucose [6, 8]. Glycated hemoglobin is measured primarily to identify average plasma glucose concentrations over prolonged periods of time and HbA1c has a strong predictive value for diabetes complications [9–12]. The target HbA1c level is <6.5% (48 mmol/mol) and this concentration is recommended as a diagnostic indicator of disease control and the effectiveness of therapy. The test should be repeated every 3 months until the target value is reached, and every 6 months thereafter [13–17]. If dietary treatment for DM is not effective, then pharmacological treatment is usually initiated. Unless contraindicated, metformin is recommended as a first line therapy in addition to diet and exercise.

National guidelines for treating DM in the Balkans generally follow European guidelines; however, the rate of routine HbA1c measurements in type 2 DM patient care among practitioners in the Balkans is uncertain. The current study was undertaken to estimate the rate of HbA1c measurement and the level of disease control in patients with type 2 DM in the Balkans, and to evaluate if providing a cost free and simple way to measure HbA1c levels leads to the more aggressive treatment of patients who have poor disease control. The primary objective of this study was to determine the rate of HbA1c testing in patients with type 2 DM among general practitioners (GPs) and diabetes specialists, and to estimate adherence to treatment guidelines. The secondary objective was to establish whether HbA1c testing improves adherence to treatment guidelines.

## Patients, materials and methods

The study was performed as a multicenter study in the Balkan countries, represented by Croatia, Slovenia, Serbia, Romania, and Bulgaria. This study was performed as an observational, non-interventional, and cross-sectional study. It was performed over 3‑month periods, in 2013 and the beginning of 2014 (Table 1, available online).

**Table 1:** Study flow chart

	Approximate duration in months
Planning and obtaining approval from authorities (ethical and regulatory agency approval)	2
Investigators meeting, training for doctors involved in study procedures, including the use of measurement devices	1
Recruiting patients, obtaining informed consent and collecting data	1
Collecting of case report forms (CRF)	1
Data analysis	1

### HbA1c measurement

Physicians were provided with HbA1c measuring devices (Siemens, Erlangen, Germany) for the purpose of the study [18].

### Diagnostic criteria

Diabetes was defined according to the 2006 WHO diagnostic criteria as fasting plasma glucose ≥7.0 mmol/l (126 mg/dl) or 2‑h plasma glucose ≥11.1 mmol/l (200 mg/dl) [3].

### Physicians

The participating physicians represented different regions of the Balkan countries. The target was to enrol equal numbers of general practitioners and specialists in each country, except in Romania, where only specialists were enrolled due to the fact that patients with type 2 diabetes are treated only by internists/diabetologists. A total of 181 physicians participated in this study of whom 79 were GP/general medical specialists and 102 were internists/diabetologists. All participating physicians attended several training meetings and were certified before recruitment. The physicians were divided into three groups according to HbA1c measurement frequency. At the end of the study, physicians were asked to provide feedback as to whether the HbA1c level results of their patients were as expected, better than expected, or worse than expected.

### Patients

Eligible patients with type 2 DM were adults (≥18 years of age) treated with oral antihyperglycemic agents or insulin at least 6 months before enrollment. A total of 1853 patients were enrolled, 479 patients treated by GP and 1331 regularly seen by diabetes specialists. The patients were recruited during regular office visits after signing an informed consent form. The population living in the study area was genetically a relatively uniformly Caucasian population, and no different ethnic groups were expected to be living there. The total population of the Balkans is 59 million of whom 34 million are adults between 27 and 69 years of age. Bearing in mind that the average prevalence of diabetes in the region was 7.6% in 2013 according to the IDF, the rough estimate is that there were approximately 3 million adults with diabetes in the region at the time the study was conducted [1]. Patient history data were taken along with HbA1c measurements. Fingerprick testing of HbA1c was performed on site [18]. Diabetes therapy on the visit date and any change in therapy on the same date, possible referral to a specialist, prior medication history, and records of prior medication taken by the subject within the past 6 months before the study were all noted.

### General informed consent

General informed consent was documented with a consent form signed and dated by both the subject or the subject’s legal representative and the person conducting the consent discussion. A copy of the signed and dated consent form was given to the subject before their participation in the study. Informed consent adheres to institutional review board/ethics review committee (IRB/ERC) requirements, applicable laws and regulations, and Sponsor requirements.

### Statistical analysis

All statistical analysis was performed using Statistical Package for the Social Sciences (SPSS) version 17.0 for Windows (SPSS, Chicago, IL). The statistical methods used for the requirements of the study were tests of the statistical significance of differences between proportions and Student’s t‑test. According to the power analysis, each subsample should have at least 21 physicians and 80 patients to have sufficient power (0.9) for the desired effect size and significance level (0.05). The study was designed to have 90% power to detect a difference of 5%. A *p*-value of 0.05 was considered statistically significant. Although the patients were recruited using a two-step procedure, the recruiting physician was not used as a random factor in the analysis The preliminary variance components analysis showed that the recruiting site does not have a significant impact on primary outcome (HbA1c level), showing no significance for either recruiting physician or the interaction with country or GP/specialist. This is probably due to both the relatively low number of patients per doctor [10] and the fact that the patients are not necessarily treated by the recruiting doctor.

## Results

### Patient baseline characteristics

**Fig. 1:**
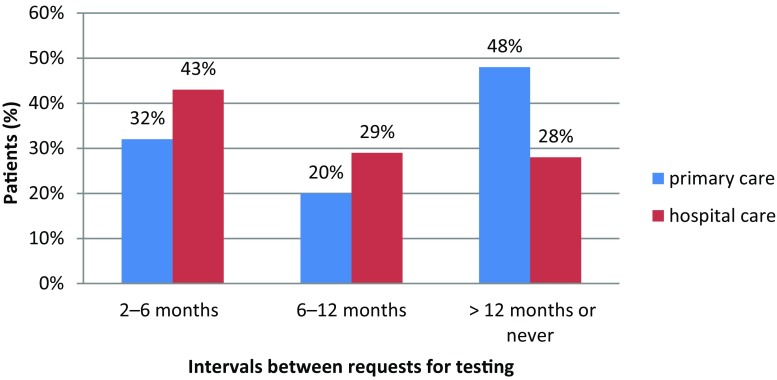
Relationship between HbA1c testing intervals and point of care

All patients were adults between 20 and 92 years of age (average age 63.5 ± 10.7 years). They had been diagnosed with type 2 DM for 1–61 years (average 8.9 ± 7.1 years) and 51% were male. Of the patients 35% had achieved the target HbA1c value of <6.5% (48 mmol/mol), while 33% had HbA1c levels >7.5% (58 mmol/mol). Measurement of HbA1c was found to be quite infrequent, with only 40% of patients having their hemoglobin measured at least every 6 months and as many as 34% less than every 12 months or never (Fig. 1).

### Glycemic control

The results of the present study showed that the level of glycemic control in the region was poor, with an average HbA1c level of 7.3% ± 1.5 (56 mmol/mol). The difference between the proportion of well-controlled patients was not statistically significant between doctors who do and do not have access to HbA1c measurement, nor were they statistically significant between GPs and specialists (*p* = 0.87 and 0.099, respectively). The difference between the proportion of well-controlled patients of doctors who measured HbA1c often and of those who do not was not statistically significant either (*p* > 0.05).

### HbA1c measurement

**Fig. 2:**
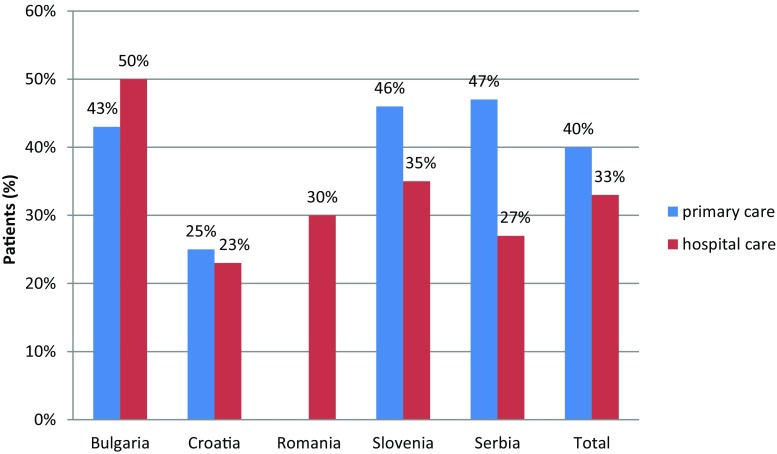
Patients who have achieved adequate glycemic control (HbA1c < 6.5%)

It is important to note that the proportion of patients with adequate glycemic control ranged from 23–50% (Fig. 2). It was significantly lower in Croatia, as compared with Serbia, Slovenia, and Bulgaria, and was significantly higher in Bulgaria than any other Balkan country except for Serbia, where the difference is not statistically significant. The proportion of patients with adequate glycemic control was significantly lower in patients of specialists across the entire sample (*p* = 0.016). These differences are not significant for Bulgaria, Croatia, or Serbia (*p* = 0.4, 0.6 and 0.2, respectively) but are significant for Slovenia (*p* = 0.001).

### Frequency of measurement

**Fig. 3:**
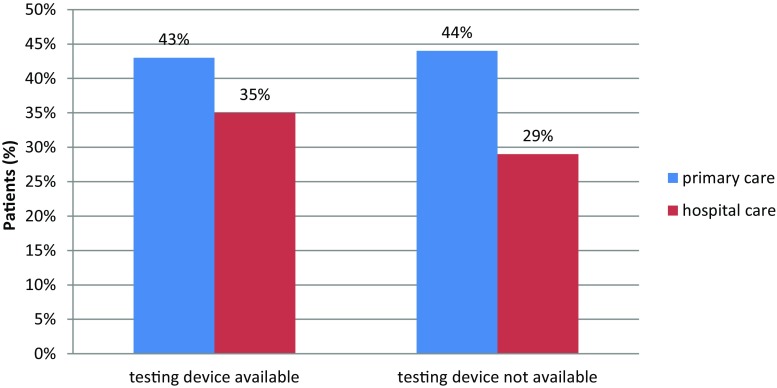
Relationship between availability of point-of-care HbA1c testing device and adequate glycemic control achievement (HbA1c < 6.5%)

**Fig. 4:**
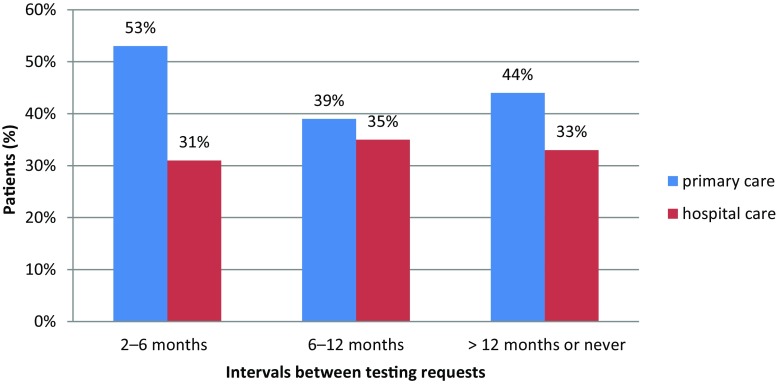
Relationship between HbA1c testing interval and adequate glycemic control achievement (HbA1c < 6.5%)

The frequency of measurement was also found to be higher in patients regularly monitored by specialists (43% vs. 32% of patients monitored at least every 6 months, *p* < 0.01) (Figs. 1, 3 and 4).

### Diabetes therapy

**Fig. 5:**
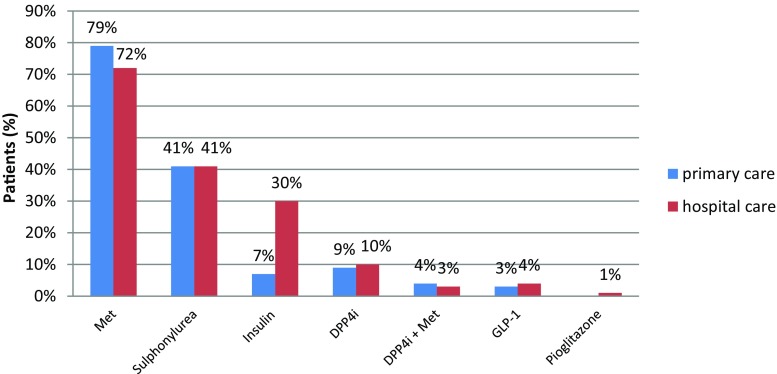
Initial diabetes therapy (*Met* metformin, *DPP-4i* dipeptidyl peptidase-4 inhibitor, *DPP-4i* *+ Met* dipeptidyl peptidase-4 inhibitor + metformin, *GLP-1* glucagon-like peptide-1 receptor agonists)

The most frequent therapy was shown to be metformin monotherapy (28% of patients), followed by a combination of metformin and sulphonylurea (23%) regardless of a low level of disease control in those patients (Fig. 5). Furthermore, adequate disease control was achieved in 55% of patients undergoing metformin monotherapy and in 32% undergoing combined therapy.

Patients treated with metformin had established better disease control; however, 40% of them still showed no improvement. Insulin was also quite prevalent, and was the only drug prescribed almost exclusively by specialists; however, patients on insulin were very poorly controlled. The difference between metformin and insulin prescription was statistically significant between GPs and specialists (*p* = 0.007 and *p* < 0.001, respectively). The use of the drug showed a similar general pattern across the countries in both subsamples.

### Changes in therapy and interventions

**Fig. 6:**
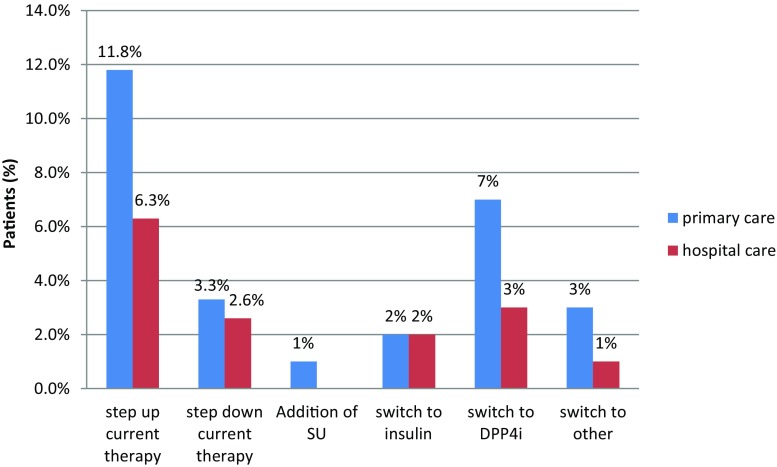
Therapeutic intervention approach in patients with inadequate glucose control (*SU* sulphonylurea, *DPP-4i* dipeptidyl peptidase-4 inhibitor)

**Fig. 7:**
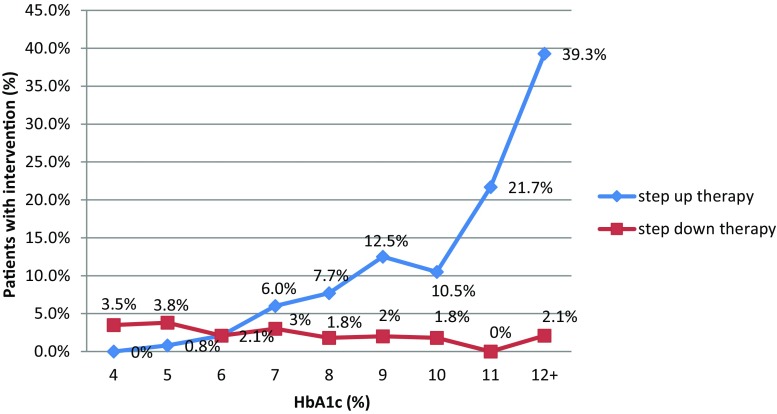
Relationship between therapeutic intervention approach by stepping up or stepping down therapy and HbA1c measure at follow-up visit

Measurement of HbA1c in physician’s offices resulted in only a small percentage of therapy changes or interventions in patients with an HbA1c level ≥6.5% (48 mmol/mol); therapy was unchanged in 91% of cases, stepped up in only 7.2% of cases, adding sulphonylurea to metformin, prescribing a dipeptidyl peptidase-4 (DPP-4) inhibitor, introducing insulin or other drugs (Figs. 6 and 7). Another interesting fact noted was that therapy was stepped down for 2% of patients by switching from combined metformin and sulphonylurea therapy to monotherapy (Fig. 6). Finally, doctors were mostly not surprised (79%) or were pleasantly surprised (11%) by the patients HbA1c results and 53% of doctors stated that they would monitor HbA1c more frequently in the future.

A stepwise multivariate regression across patient characteristics (age, time since diagnosis, treated by GP or specialist) and manner of disease monitoring (measuring blood glucose using glucometer at home or measuring HbA1c) and frequency of measurement (number of blood glucose measurements or time since last HbA1c measurement) yielded a statistically significant model with the major predictor variable impact of type-2 DM time since diagnosis (significance <0.001, predictor importance 0.608) and the use of blood glucose as a measurement tool (significance <0.001, predictor importance 0.240). The usage and the frequency of HbA1c measurement was not shown to significantly influence DM control in the region.

## Discussion

People with Type 2 DM can remain undiagnosed for many years, unaware of the long-term damage caused by the disease. The struggle for tight glycemic control results in large blood glucose fluctuations over time. These fluctuations are the measurable result of the action of a complex dynamic system that is influenced by many internal and external factors, including the timing and amount of insulin and other drug therapies, diet, physical activity. [19].

### Routine HbA1c measurement

The level of HbA1c provides a reliable measure of chronic glycemic control without the need for a fasting or timed sample. Routine measurement of HbA1c has remained the standard biomarker and the gold standard for monitoring glycemic control in patients with diabetes [8, 19]. Evidence shows that having an HbA1c result at the time of a doctor’s visit is beneficial [11]. According to previous studies HbA1c is also a practical and convenient tool for screening undiagnosed diabetes in a routine health check-up [7, 20–22, 27].

The rate of routine measurements of HbA1c in Type 2 DM patient care in the Balkans is uncertain. The results of a study performed in Croatia in 2011 implied poor glycemic control [16]. The level of glycemic control in patients is unknown both in Croatia and in other Balkan countries. According to the results of the study, measurement of HbA1c is generally quite low, especially in GP patients, and is lower than 50% in all countries. Specialists measure HbA1c more often in Croatia and Serbia, while in Slovenia and Bulgaria they measure it just as rarely as GPs. In general 50% of GP patients and 41% of specialist patients did not have HbA1c measurements taken in over 1 year. Significantly, if the patient is seen more frequently by a specialist, it is more likely that a current HbA1c measurement is available. A higher frequency of measurement by specialists is likely due to better access to measurement in hospitals and specialists’ higher level of education.; however, not even specialists seem to measure HbA1c for all patients. The significantly lower proportion of patients at specialists with adequate glycemic control can be explained by the referral of more difficult patients from GPs to specialists for therapy modification and more strictly controlled treatment. Existing data show that real-time HbA1c estimation could increase patient motivation to improve diabetes control [19].

### Cut-off value

Although the results of HbA1c measurement in this study were poor (only 35% of patients had HbA1c < 6.5%), less strict thresholds would only moderately increase the proportion of well-controlled patients but still leave a considerable proportion poorly controlled, regardless of the criteria used. It is incumbent on the clinician to know when HbA1c results should be questioned, such as when the value is discordant from the patient’s self-monitoring blood glucose values, or when there has been an acute change in glycemia, such as recent treatment with glucocorticoids [8].

### Therapy

Patients on metformin were better controlled but more than 40% were not. Noticeably, patients on insulin were very poorly controlled, implying the late introduction of insulin; however, it was noticed that therapy was stepped up only for a small number of patients (5–7%).

### Self-management

Self-management is achievable through adherence to professional advice regarding diet, exercise, and medication. The effectiveness of structured self-monitored blood glucose testing has an empowerment effect on the patient and results in better metabolic control in people with type 2 DM [23]. The more active participation of patients in self-care management produces better metabolic outcomes and better compliance with treatment protocols [23]. A study performed in the Balkans included only adult patients. A study on elderly type 2 DM patients showed that HbA1c is a relevant tool for assessing patient glycemic control and adherence [24]. Complimentarily, HbA1c measured at gestational DM (GDM) diagnosis may be a useful tool for identifying GDM patients at highest risk of developing postpartum abnormal glucose [25]. Considering the fact that weight-related problems are on the rise in many populations worldwide, diabetes experts are still leading the battle against DM. The progression of diabetes can also be delayed by intensive intervention [26].

### Study strengths and limitations

The strengths of the study are the comprehensive approach to its design as an observational, noninterventional, and cross-sectional study, the highly-structured protocol, and the set of statistical analyses to determine the effects of protocol completion. There were no major limitations to this study. The lack of a control is a minor limitation of this observational study; however, the design is appropriate for the intended purpose of the study.

## Conclusion

Type 2 DM is a noteworthy preventable disease and an increasing public health problem [26]. Effective management requires partnership between diabetes patients and health professionals. The appropriate use of structured self-monitoring of blood glucose significantly improves glycemic control and facilitates adherence to recommendations about nutrition and physical activity as well as more timely and aggressive treatment changes without decreasing general wellbeing [28].

The main finding of this study is that the provision of an easy to use and cost-free HbA1c measurement device does not positively influence treatment patterns of patients with type 2 DM in the Balkans. In this age of modern technology, online monitoring, and available testing it is unacceptable that less than 50% of diabetes patients are well-controlled. Emerging technologies have created continuous glucose monitoring systems that allow frequent, real time glucose measurements, which may possibly be superior to oral glucose tolerance test (OGTT) and HbA1c measurement [29]. The move towards HbA1c-based diagnosis has obviously generated controversy. Unfortunately, diabetes is still not under control in the Balkans. More studies are necessary to explore the reasons for this phenomenon. The usage and frequency of HbA1c measurement was not shown to significantly influence DM control in the region. Nevertheless, the results that this study has produced show that HbA1c measurement should be performed strictly according to the guidelines.
